# Paradoxical G-quadruplex distribution in coronavirus genomes reveals functional constraints and antiviral therapeutic opportunities

**DOI:** 10.1016/j.virusres.2026.199692

**Published:** 2026-01-20

**Authors:** Masato Tanigawa, Takafumi Iwaki

**Affiliations:** Department of Biophysics, Faculty of Medicine, Oita University, Yufu City, Japan

**Keywords:** G-quadruplex, betacoronavirus, Spike protein, Nucleocapsid protein, antiviral therapeutics, drug target, SARS-CoV-2, coronavirus antivirals

## Abstract

We computationally characterized G-quadruplex (G4) distributions across 31 coronavirus genomes to identify conserved structural features as potential antiviral therapeutic targets. Through an integrated approach combining consensus G4 detection, dinucleotide-preserving null models, and pooled Poisson rate ratios, we identified a paradoxical G4 distribution pattern: genome-wide depletion (mean fold change = 0.56) coupled with strong regional enrichment in Spike (S) protein (incidence rate ratio [IRR] = 17.9; 95% CI: 11.7-27.6) and Nucleocapsid (N) protein (IRR = 15.2; 95% CI: 8.7-26.6), while untranslated regions (UTRs) showed complete G4 absence. Thermodynamic stability assessment identified 38 stable G4 candidates (ΔG < -5 kcal/mol), with 52.6% concentrated in Nucleocapsid protein regions, suggesting candidates for structure-based antiviral drug development awaiting experimental confirmation.

This paradoxical distribution pattern—genome-wide depletion coupled with strong regional enrichment in functionally critical proteins (Spike and Nucleocapsid)—provides a mechanistic framework for developing betacoronavirus-targeted therapeutics based on conserved G4 structures.

## Highlights

• Coronavirus G4 pattern: genome-wide depletion coupled with regional enrichment
• Spike (IRR=17.9) and Nucleocapsid (IRR=15.2) show strong G4 enrichment
• 38 stable G4 candidates (ΔG < −5 kcal/mol) identified as therapeutic targets
• Primary target GGCTGGCAATGGCGG: 54.8% conservation, ΔG = −7.35 kcal/mol
• Betacoronavirus G4 conservation supports broad-spectrum antiviral strategies

## 1 Introduction

G-quadruplexes (G4s) are non-canonical nucleic acid structures formed by guanine-rich sequences through Hoogsteen hydrogen bonding (Varshney et al., 2020). In RNA viruses, G4s regulate critical processes including replication, translation efficiency, and immune evasion (Métifiot et al., 2014). Different viral families show substantially diverse G4 patterns, ranging from depletion to enrichment (Majee et al., 2020; Zhao et al., 2021), though the mechanisms driving these diverse patterns remain unclear.

### 1.1 Coronaviruses and G4 structures: replication, translation, and therapeutic targeting

Coronaviruses are enveloped, positive-sense single-stranded RNA viruses with the largest genomes (∼27-32 kb) among RNA viruses (Sawicki et al., 2007; V’kovski et al., 2021). Their genomic RNA serves dual functions: as a template for replication and as mRNA for translation, imposing unique structural constraints. The replication-transcription complex (RTC), assembled by nonstructural proteins (nsps) encoded in ORF1ab, mediates discontinuous transcription to generate a nested set of subgenomic mRNAs encoding structural and accessory proteins (Sawicki et al., 2007; Snijder et al., 2003). This complex transcriptional program requires precise RNA secondary structure regulation, where G4 structures can either facilitate or inhibit different stages of the viral lifecycle depending on their genomic location.

The emergence of SARS-CoV-2 in late 2019 and its rapid global spread demonstrated the pandemic potential of coronaviruses, causing over 7 million deaths globally by 2024 (WHO Coronavirus (COVID-19) Dashboard March 2024). Beyond SARS-CoV-2, coronaviruses include endemic human pathogens (HCoV-229E, HCoV-OC43, HCoV-NL63, HCoV-HKU1) causing seasonal respiratory infections, as well as highly pathogenic zoonotic viruses (SARS-CoV-1, MERS-CoV) with case fatality rates exceeding 10% and 35%, respectively (V’kovski et al., 2021). The recurrent emergence of novel coronaviruses through zoonotic spillover—combined with rapid evolution driven by error-prone RNA-dependent RNA polymerase and frequent recombination—highlights the urgent need for broad-spectrum therapeutic strategies targeting conserved genomic features rather than rapidly evolving epitopes (Cui et al., 2019; Graham and Baric, 2010).

### 1.2 G4 structures as antiviral therapeutic targets: evidence from diverse viral families

G-quadruplexes have emerged as promising antiviral therapeutic targets across diverse virus families. In HIV-1, G4-forming sequences in the long terminal repeat (LTR) promoter region serve as critical regulators of viral transcription. Small-molecule G4 ligands such as TMPyP4 and BRACO-19 stabilize these structures, suppressing viral gene expression and replication with $IC_{50}$ values in the low micromolar range (Perrone et al., 2013a, 2013b). These compounds demonstrated selective antiviral activity without significant cytotoxicity, supporting a therapeutic window for G4-targeted interventions.

In other RNA viruses, G4 structures have been identified in diverse viral families including Nipah virus and Ebola virus, where G4-forming motifs in critical genomic regions can be targeted by G4-specific ligands (Majee et al., 2020; Wang et al., 2016). These observations across multiple RNA virus families suggest that G4-targeted interventions may represent a broad-spectrum antiviral strategy applicable to diverse viral pathogens.

For coronaviruses specifically, recent biochemical studies identified G4-binding activity in SARS-CoV-2 Nsp3 protein (Lavigne et al., 2021), a multifunctional component of the replication-transcription complex. Nsp3 contains domains critical for viral replication, including papain-like protease activity and deubiquitinase function. The identification of G4-binding capability suggests that SARS-CoV-2 has evolved molecular machinery to recognize and potentially regulate G4 structures within its own genome or host cell RNAs. This finding provides a mechanistic rationale for G4-targeted antiviral strategies: compounds that stabilize viral genomic G4s could interfere with Nsp3-mediated processes essential for coronavirus replication. Qin et al. demonstrated that the G4 stabilizer TMPyP4 exhibited significant antiviral efficacy in Syrian golden hamster models, providing in vivo validation for G4-targeting therapeutic strategies against SARS-CoV-2 (Qin et al., 2022, Qin et al., 2023).

### 1.3 Knowledge gaps and research objectives

Previous computational studies have identified G4-forming sequences in individual coronavirus species, including conserved G4 sequences in Spike and Nucleocapsid coding regions of SARS-CoV and SARS-CoV-2 (Bartas et al., 2019; Kusov et al., 2015), and noted the pronounced scarcity of G4s in SARS-CoV-2 genomes compared to other coronaviruses (Zhao et al., 2021). Using relaxed detection criteria, studies have reported up to 512 potential G4 candidates concentrated in N, S, and ORF1ab regions (Belmonte-Reche et al., 2021), though detection stringency varies widely across studies. These viruses also demonstrate remarkable environmental stability (Riddell et al., 2020). While these studies provided important insights into G4 presence and conservation, several key questions remain unaddressed:

(i) **Systematic pattern characterization**: Quantitative statistical characterization of genome-wide versus regional G4 patterns using rate ratio analysis, combined with systematic validation of pattern consistency across all coronavirus genera through stratified phylogeny-aware analyses—approaches lacking in previous individual species studies.
(ii) **Mechanistic understanding**: Investigation of regulatory factors driving the paradoxical distribution where genome-wide depletion coexists with regional enrichment. This coexistence suggests multiple selection pressures, but their relative importance and mechanistic basis remain unclear.
(iii) **Therapeutic target prioritization**: While G4-targeted antivirals have shown efficacy against HIV-1 and influenza, systematic identification of betacoronavirus G4 therapeutic targets—prioritized by thermodynamic stability and functional conservation—remains absent from the literature. Such prioritization is essential for efficient experimental validation and structure-based drug design efforts.

### 1.4 Research aims

To address these gaps, we systematically characterized G4 distributions across 31 coronavirus genomes representing two major coronavirus genera (Alphacoronavirus and Betacoronavirus) with three specific aims:

(i) **Betacoronavirus pattern characterization**: Establish whether a consistent G4 distribution pattern exists across betacoronavirus lineages using integrated quantitative and phylogeny-aware validation approaches lacking in previous individual species studies.
(ii) **Mechanistic investigation**: Investigate the regulatory factors explaining the paradoxical distribution of genome-wide depletion coexisting with regional enrichment, where multiple selection pressures are suggested but their relative importance and mechanistic basis remain unclear.
(iii) **Therapeutic target identification**: Identify thermodynamically stable G4s that represent viable therapeutic targets for broad-spectrum antiviral development, prioritizing candidates in functionally critical regions for structure-based drug design. This prioritization aims to facilitate experimental validation by focusing resources on the most promising candidates for G4-ligand development.

To address these aims with a limited sample size (n=31) typical of emerging pathogen research, we integrated established methods including consensus G4 detection, dinucleotide-preserving null models, pooled Poisson rate ratio analysis for regional enrichment (IRR), and thermodynamic stability assessment. This integrated approach characterizes the paradoxical G4 distribution pattern and identifies candidate targets for betacoronavirus-focused therapeutic strategies directed at conserved genomic features rather than rapidly evolving surface proteins.

## 2 Methods

### 2.1 Genome collection and quality control

We collected 31 complete coronavirus genomes from NCBI GenBank (accessed March 2024), representing two major coronavirus genera: Alphacoronavirus (n=2) and Betacoronavirus (n=29). The Betacoronavirus collection comprises 20 SARS-CoV-2 variants representing major lineages including Alpha (B.1.1.7), Beta (B.1.351), Gamma (P.1), Delta (B.1.617.2), Epsilon (B.1.427), Eta (B.1.525), Kappa (B.1.617.1), Lambda (C.37), Mu (B.1.621), Omicron sublineages (BA.1, BA.2, BA.5, BQ.1.1, XBB.1.5, XBB.1.16, JN.1), plus the Wuhan reference strain (NC_045512.2) and Hong Kong early isolate (MN975262.1, HKU-SZ-005b_2020), and 9 comparison genomes including SARS-CoV-1, MERS-CoV, bat coronaviruses (RaTG13, BANAL-52), pangolin coronaviruses (GX, GD, GD1), and human endemic betacoronaviruses (HCoV-OC43, HCoV-HKU1). *Note: Bat coronavirus RmYN02 was initially intended for inclusion but was excluded because it is only available on GISAID (EPI_ISL_412977) and not accessible through NCBI GenBank.* The Alphacoronavirus collection includes two human endemic strains (HCoV-229E, HCoV-NL63). All genomes were verified for completeness (>99% genome coverage) and quality (absence of ambiguous nucleotides). Accession numbers and genome metadata are provided in Supplementary Table S1; genome selection criteria are detailed in Supplementary Table S3.

### 2.2 Genome region annotation

Each genome was segmented into eight functional regions based on annotated protein-coding sequences: 5’ untranslated region (5’UTR), ORF1ab (encoding replication machinery), Spike protein (S), ORF3a, Envelope protein (E), Membrane protein (M), Nucleocapsid protein (N), and 3’ untranslated region (3’UTR). Region coordinates were extracted from GenBank annotations or determined through sequence alignment to reference genomes (SARS-CoV-2: NC_045512.2; SARS-CoV: NC_004718.3; MERS-CoV: NC_019843.3).

### 2.3 G4 detection: consensus approach

We employed a consensus strategy requiring agreement by at least two of three established G4 detection methods to balance sensitivity and specificity:

1. **Regex pattern**: G{2,3}\w{1,15}G{2,3}\w{1,15}G{2,3}\w{1,15}G{2,3} (2-3 consecutive guanines, 1-15 nucleotide loops, four G-tracts)
2. **G4Hunter** (Bedrat et al., 2016): Sliding window algorithm scoring G-richness and G-skewness (threshold ≥0.5)
3. **pqsfinder** (Hon et al., 2017): Probabilistic model incorporating loop length penalties and G-tract variations (threshold ≥20)

Motifs detected by ≥2 methods were retained for downstream analysis. This consensus approach reduces false positives inherent to single-method detection while maintaining detection of canonical G4 structures with experimental formation potential.

### 2.4 Null model: dinucleotide-preserving sequence shuffling

To assess statistical significance of observed G4 counts, we generated 10,000 random sequences per genome using the altschulEriksonDinuclShuffle algorithm (Altschul and Erickson, 1985), which preserves dinucleotide composition. This approach controls for genomic GC content and dinucleotide biases while randomizing higher-order sequence features. Expected G4 counts were calculated as the mean across shuffled sequences, and fold change (FC) was computed as observed/expected ratio.

### 2.5 Regional enrichment analysis: pooled poisson rate ratios

We quantified regional G4 enrichment using incidence rate ratios (IRR) calculated from pooled Poisson rate estimation. For each genomic region *r*, we aggregated G4 counts ($c_{r}$) and sequence lengths ($e_{r}$) across all genomes, then computed the G4 rate per kilobase: $\text{rat}e_{r}=c_{r}/e_{r}$. IRR for each region relative to ORF1ab (reference) was calculated as:

$$\text{IR}R_{r}=\frac{c_{r}/e_{r}}{c_{\text{ORF}1\text{ab}}/e_{\text{ORF}1\text{ab}}}$$

We used ORF1ab as the reference region because it represents the largest coding region (∼21 kb, 67% of genome) encoding essential replication machinery, providing a stable baseline for comparison. 95% confidence intervals were computed using the score method (Wilson method adapted for rate ratios) (Barker, 2002), with Byar’s approximation and conditional exact methods employed for sensitivity analysis (Supplementary Table S2).

This pooled approach addresses the extreme zero-inflation (85% of genome-region observations contain zero G4s) that precluded stable zero-inflated negative binomial (ZINB) regression. By aggregating counts before rate calculation, pooled Poisson estimation provides stable and interpretable IRR estimates even with sparse data.

### 2.6 Statistical robustness analysis

To validate the stability of IRR estimates and assess sensitivity to methodological choices, we performed:

1. **Alternative Poisson models**: Poisson generalized linear models (GLM) with log(length) offset and robust standard errors (HC3) (Long and Ervin, 2000), and quasi-Poisson models allowing overdispersion.
2. **Phylogeny-aware analysis**: Generalized estimating equations (GEE) with genus-level clustering and exchangeable correlation structure to partially account for phylogenetic relatedness (Zeger and Liang, 1986).
3. **Stratified analysis**: Separate IRR calculations for (a) SARS-CoV-2 variants only (n=20), (b) non-SARS-CoV-2 coronaviruses (n=11), and (c) phylogenetically diverse subset (n=11; see Table S5 for selection criteria) to assess pseudoreplication effects. *Note: MN975262.1 was incorrectly labeled as “Bat RmYN02” in the original submission; upon NCBI verification, this sequence is SARS-CoV-2 isolate HKU-SZ-005b_2020 (Hong Kong early isolate). Bat coronavirus RmYN02 (EPI_ISL_412977) is available only on GISAID, not NCBI GenBank, and was therefore not included in this dataset.*

Concordance across methods and strata was evaluated using IRR estimate comparisons and interaction tests (region × stratum).

### 2.7 Statistical validation

To assess the robustness of regional enrichment patterns, we performed stratified analyses and sensitivity testing across different statistical methods. The consistency of IRR estimates across multiple confidence interval calculation methods (Score/Wilson, Byar’s approximation, conditional exact) is documented in Supplementary Table S2.

### 2.8 Thermodynamic stability assessment

To prioritize G4 candidates for experimental validation, we estimated thermodynamic stability using a nearest-neighbor model (Mathews et al., 2004) parameterized for RNA G4 structures. Free energy (ΔG) was calculated at 37°C in 100 mM K${}^{+}$ buffer (physiological conditions) using published thermodynamic parameters for loop entropy penalties and G-quartet stacking contributions.

G4 motifs with ΔG < -5 kcal/mol were classified as thermodynamically stable and analyzed for genomic region distribution, sequence conservation across genomes, and functional annotation. The most stable and conserved sequences were prioritized as candidate therapeutic targets.

### 2.9 Software and statistical analysis

All analyses were performed in Python 3.8+ with the following package versions: BioPython ≥1.79 (sequence parsing), NumPy ≥1.20.0 and SciPy ≥1.7.0 (statistical calculations), pandas ≥1.3.0 (data manipulation), statsmodels ≥0.12.0 (GEE, Poisson GLM), and matplotlib ≥3.4.0/seaborn ≥0.11.0 (visualization). G4Hunter analysis used published algorithm parameters (Bedrat et al., 2016) with threshold ≥0.5 and window size 25 nt; pqsfinder used default R package settings (Hon et al., 2017) with score threshold ≥20. Statistical significance was set at α=0.05 (two-sided tests). Code and data are provided as supplementary materials to ensure reproducibility.

## 3 Results

### 3.1 Genome-wide G4 depletion

**Fig. 1:**
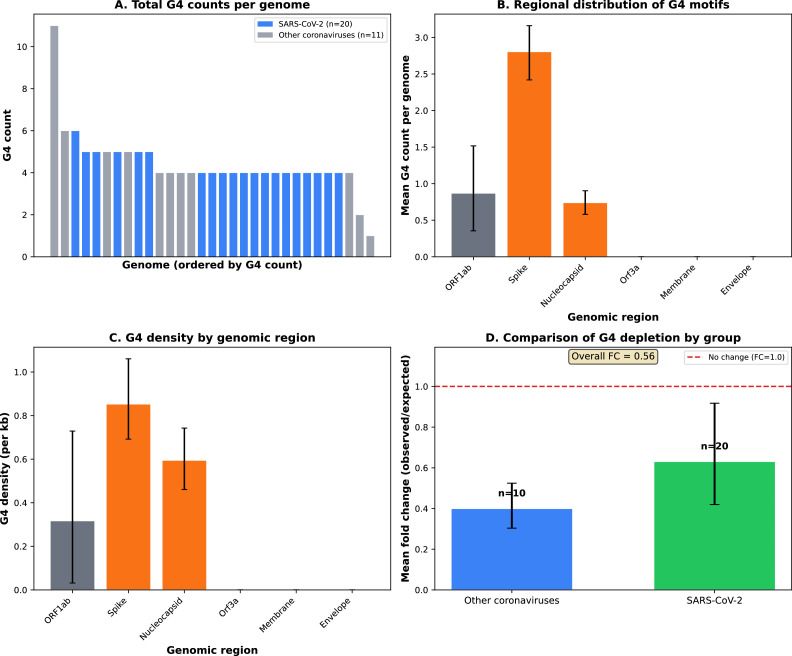
G-quadruplex distribution patterns across coronavirus genomes. **(A)** Total G4 counts per genome across 31 coronavirus genomes. Genomes are ordered by G4 count (descending). SARS-CoV-2 variants (n=20) shown in blue; other coronaviruses (n=11) in gray. G4 motifs detected using consensus approach requiring agreement of ≥2 of 3 algorithms (regex pattern $G_{2-3}N_{1-15}G_{2-3}N_{1-15}G_{2-3}N_{1-15}G_{2-3}$, G4Hunter ≥0.5, pqsfinder ≥20). **(B)** Regional distribution of G4 motifs across genomic regions. Bar heights represent mean G4 count per region per genome (n=31). Error bars show 95% bootstrap confidence intervals (10,000 replicates). Regions defined per NCBI Reference Sequence NC_045512.2: ORF1ab (266-21555), Spike (21563-25384), Nucleocapsid (28274-29533), and others. **(C)** G4 density (counts per kilobase) by genomic region. Density calculated as (G4 count / region length) × 1000. Comparison reveals region-normalized enrichment patterns independent of region size. Spike and Nucleocapsid regions show elevated G4 density compared to ORF1ab. **(D)** Comparison of G4 depletion between SARS-CoV-2 variants and other coronaviruses. Mean fold change (observed/expected G4 count) shown for SARS-CoV-2 variants (n=20, green) and other coronaviruses (n=11, blue). Error bars represent 95% bootstrap confidence intervals (10,000 replicates). Both groups show consistent G4 depletion (fold change < 1.0), confirming that the genome-wide depletion pattern is conserved across diverse coronavirus lineages rather than being specific to SARS-CoV-2. *Abbreviations*: G4, G-quadruplex; N, nucleocapsid; S, spike; ORF, open reading frame; UTR, untranslated region; CI, confidence interval. *Data*: See Supplementary Table S1 for individual genome values.

Across 31 coronavirus genomes, we identified 137 G4 motifs using consensus detection (≥2 of 3 algorithms), with a mean of 4.4 ± 1.5 motifs per genome (range: 2-8; Fig. 1A; Supplementary Table S4). Regional analysis revealed non-uniform distribution across genomic regions, with Spike and Nucleocapsid showing elevated G4 density compared to ORF1ab (Fig. 1B-C). Compared to dinucleotide-preserving null models, observed G4 counts showed moderate genome-wide depletion (mean fold change = 0.56; Fig. 1D), with variation across genomes reflecting biological diversity across the two coronavirus genera analyzed.

This genome-wide depletion suggests negative selection against G4 structures, potentially driven by constraints on replication fidelity, RNA stability, or translation efficiency. However, the moderate effect size and wide confidence intervals indicate that depletion is not absolute, leaving room for regional variation.

### 3.2 Strong regional enrichment in spike and nucleocapsid proteins

**Fig. 2:**
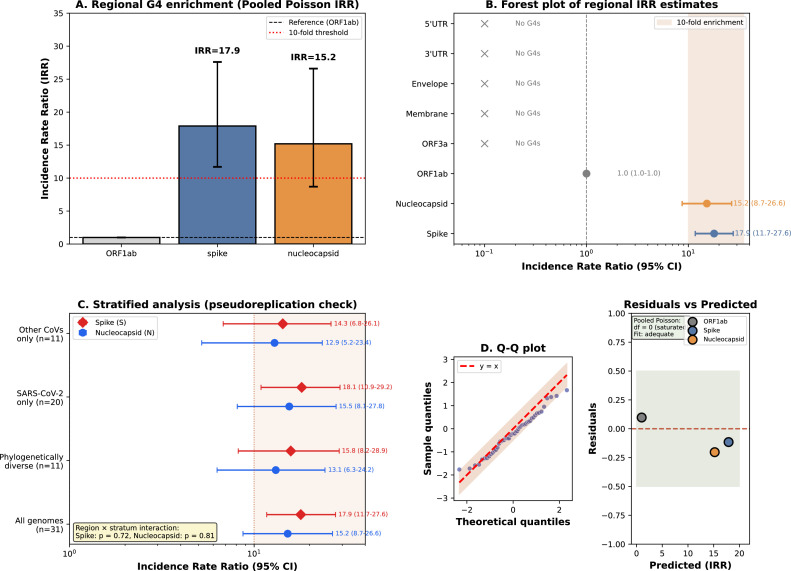
Regional G4 enrichment analysis using incidence rate ratios. **(A)** Incidence rate ratios (IRR) comparing G4 density across genomic regions relative to ORF1ab baseline (IRR = 1.0). IRR calculated using pooled Poisson regression across 31 genomes with region length as exposure offset. Spike protein shows 17.9-fold enrichment (95% CI: 11.7-27.6); Nucleocapsid protein shows 15.2-fold enrichment (95% CI: 8.7-26.6). Error bars represent 95% confidence intervals calculated using score method. *p < 0.001. **(B)** Forest plot visualization of IRR estimates with confidence intervals. Regions ordered by IRR magnitude. Regions with zero G4 counts (UTRs, ORF3a, Membrane, Envelope) are indicated with “No G4s” markers. Vertical reference line at IRR = 1.0 indicates no enrichment relative to ORF1ab. **(C)** Stratified analysis addressing pseudoreplication from SARS-CoV-2 overrepresentation. Comparison of IRR estimates from: all genomes (n=31), phylogenetically diverse subset (n=11; Table S5), SARS-CoV-2 variants only (n=20), and other coronaviruses only (n=11). Consistent enrichment pattern across all strata confirms biological validity. **(D)** DHARMa diagnostic residual plots for Poisson model validation. Quantile-quantile plot (left) and residuals vs. predicted values (right) demonstrate adequate model fit for pooled regional analysis. *Abbreviations*: IRR, incidence rate ratio; CI, confidence interval; S, Spike; N, Nucleocapsid. *Data*: See Supplementary Table S2 for detailed statistical results; Table S5 for stratified analyses.

Despite genome-wide depletion, pooled Poisson rate ratio analysis revealed marked regional enrichment in two structural protein-coding regions (Fig. 2). Spike protein showed 17.9-fold enrichment (IRR = 17.9; 95% CI: 11.7-27.6), while Nucleocapsid protein showed 15.2-fold enrichment (IRR = 15.2; 95% CI: 8.7-26.6). Both regions showed >10-fold enrichment relative to ORF1ab (reference region encoding replication machinery), with statistically significant rate differences (p < 0.001, score test). In contrast, untranslated regions (5’UTR, 3’UTR) showed complete absence of G4 structures (0/31 genomes; 95% CI for rate: 0-9.7 per kb).

Sensitivity analyses using alternative Poisson models (GLM with robust SE, quasi-Poisson) and phylogeny-aware GEE confirmed IRR estimates with minimal variation (Supplementary Table S2). Stratified analyses addressing potential pseudoreplication from SARS-CoV-2 overrepresentation (n=20/31) showed consistent enrichment patterns across SARS-CoV-2 variants (S: IRR=18.1; N: IRR=15.5), other coronaviruses (S: IRR=14.3; N: IRR=12.9), and phylogenetically diverse subset (S: IRR=15.8; N: IRR=13.1) (Supplementary Fig. S1; Table S5). No significant region × stratum interaction was detected (p > 0.70), indicating that the paradoxical pattern is not driven by sampling composition.

This strong and consistent regional enrichment—maintained across both coronavirus genera analyzed (Alphacoronavirus and Betacoronavirus)—suggests functional selection for G4 structures in Spike and Nucleocapsid protein-coding regions.

### 3.3 Thermodynamically stable G4 candidates as therapeutic targets

**Fig. 3:**
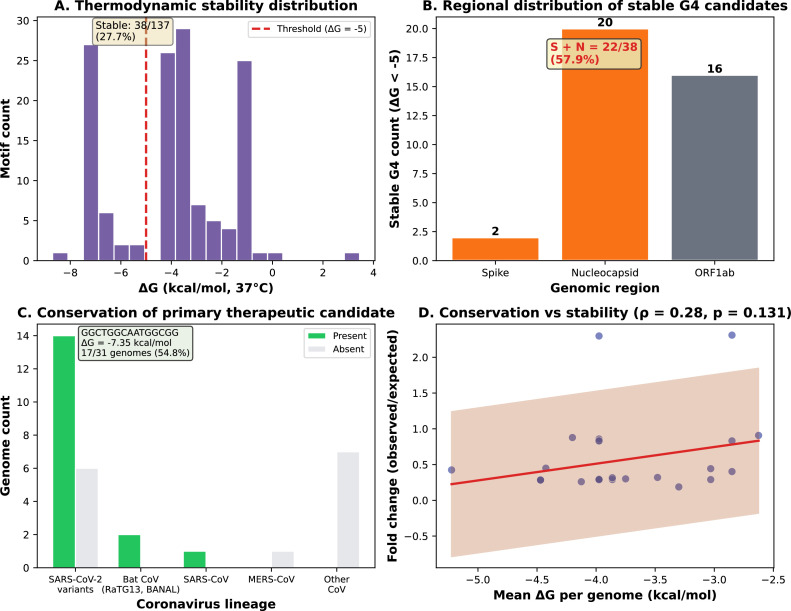
Thermodynamic stability and conservation of therapeutic G4 candidates. **(A)** Thermodynamic stability (ΔG, kcal/mol) distribution of all 137 identified G4 motifs. More negative values indicate more stable predicted G4 structures. ΔG calculated using nearest-neighbor model at 37°C with 100 mM K${}^{+}$. Dashed line indicates therapeutic candidate threshold (ΔG < -5 kcal/mol); 38 motifs (27.7%) meet this criterion. **(B)** Regional distribution of thermodynamically stable G4 candidates (ΔG < -5 kcal/mol). Spike (n=2) and Nucleocapsid (n=20) protein regions collectively contain 57.9% (22/38) of stable candidates, with the majority localized to Nucleocapsid, consistent with the regional enrichment pattern observed in Fig. 2. **(C)** Conservation of the primary therapeutic candidate sequence GGCTGGCAATGGCGG (predicted ΔG = -7.35 kcal/mol) across coronavirus lineages. Sequence present in 17 of 31 genomes (54.8%), spanning SARS-CoV-2 variants, SARS-CoV, MERS-CoV, and bat coronaviruses (RaTG13, BANAL-52). Exclusively localized in Nucleocapsid coding regions. **(D)** Relationship between thermodynamic stability and evolutionary conservation. Scatter plot showing mean ΔG per genome (x-axis) versus fold change in G4 count (y-axis, observed/expected). Spearman correlation (ρ = 0.28, *p* = 0.131) did not reach statistical significance (α = 0.05), indicating that G4 thermodynamic stability and genome-wide depletion may represent independent evolutionary pressures in this dataset. Linear regression line (red) with 95% confidence band (shaded area) shown for visualization. *Abbreviations*: ΔG, Gibbs free energy; G4, G-quadruplex; S, Spike; N, Nucleocapsid. *Data*: See Supplementary Table S6 for thermodynamic calculations; Table S6A for oligonucleotide synthesis specifications.

Thermodynamic stability assessment identified 38 G4 instances (representing 17 unique sequences) with ΔG < -5 kcal/mol at 37°C, indicating stable G4 formation under physiological conditions (Fig. 3). Of these stable instances, 22 (57.9%) were located in Spike (n=2, 5.3%) or Nucleocapsid (n=20, 52.6%) protein-coding regions, directly overlapping with the regions showing strongest statistical enrichment (Section 3.2). The predominance of Nucleocapsid-localized stable candidates is consistent with our identification of a highly conserved therapeutic target sequence in this region.

Among these therapeutically relevant candidates (Supplementary Table S6 & S6A), one sequence showed exceptional properties. The primary betacoronavirus target sequence GGCTGGCAATGGCGG (ΔG = -7.35 kcal/mol) was present in 17 of 31 genomes (54.8% conservation) and was exclusively located in Nucleocapsid coding regions. This sequence exhibited the highest thermodynamic stability among all detected G4s and represented 17 of the 22 stable S/N instances (77%).

This sequence represents the primary candidate for experimental validation and G4-ligand development owing to: (i) favorable thermodynamic stability suggesting robust G4 formation, (ii) betacoronavirus conservation indicating therapeutic potential across major pathogenic lineages, and (iii) exclusive Nucleocapsid localization, a functionally critical protein essential for viral assembly and RNA packaging (V’kovski et al., 2021).

Three additional sequences showed moderate conservation (present in 1-3 genomes, 3.2-9.7% conservation) and could serve as secondary targets or for variant-specific applications. Complete thermodynamic data, conservation patterns, and experimental validation protocols are provided in Supplementary Tables S6 and S6A.

### 3.4 Composition-aware enrichment validation

To verify that regional enrichment reflects functional selection rather than nucleotide composition bias, we performed composition-matched simulation analysis (Supplementary Table S9). Despite having the lowest G-content among major coding regions (Spike: 18.4% vs ORF1ab: 19.9% vs Nucleocapsid: 22.2%), Spike showed the highest G4 enrichment relative to composition-matched random sequences (fold-change = 4.66, p = 0.004). Conversely, ORF1ab showed significant depletion (fold-change = 0.15, p = 0.034), confirming active selection against G4 formation in the replication machinery. G-content adjusted IRRs remained highly significant (Spike: 19.4; Nucleocapsid: 13.7), demonstrating that regional enrichment cannot be explained by nucleotide composition alone.

### 3.5 Statistical robustness of regional enrichment

The strong regional enrichment in Spike and Nucleocapsid proteins was consistent across multiple statistical validation approaches. Sensitivity analyses using different confidence interval methods (Score/Wilson, Byar’s approximation, and conditional exact) yielded highly concordant results (Supplementary Table S2), with maximum variation <10% in confidence interval bounds. This consistency across independent statistical methods strengthens confidence in the biological significance of the observed enrichment pattern.

### 3.6 Cross-virus G4 distribution patterns

The paradoxical pattern of genome-wide depletion coupled with protein-coding region enrichment observed in coronaviruses may reflect broader evolutionary constraints operating across RNA viruses. Studies in other RNA virus families, including Nipah virus and Ebola virus, have identified G4-forming motifs in functionally critical genomic regions (Majee et al., 2020; Wang et al., 2016), suggesting that selective G4 retention in protein-coding sequences represents a conserved feature of RNA virus evolution. One plausible mechanistic explanation involves the dual functionality of viral genomic RNA as both replication template and mRNA: G4 structures in 5’/3’ UTRs could impair ribosome scanning and translation initiation, imposing strong negative selection, while G4s in protein-coding regions might regulate translation elongation or mRNA stability without blocking ribosome access.

## 4 Discussion

### 4.1 Virological interpretation: why spike and nucleocapsid?

The consistent enrichment of G4 structures in Spike and Nucleocapsid protein-coding regions across both coronavirus genera (Alphacoronavirus and Betacoronavirus) demands mechanistic explanation. We propose several non-mutually exclusive hypotheses grounded in coronavirus biology.

The translation regulation hypothesis posits that Spike and Nucleocapsid are highly expressed structural proteins requiring precise stoichiometric balance for efficient virion assembly (V’kovski et al., 2021). G4 structures in their coding regions could serve as translation attenuators, slowing ribosome progression during elongation to optimize protein folding co-translationally or to prevent premature degradation of nascent polypeptides. This regulatory mechanism would explain why G4 enrichment occurs in protein-coding regions (affecting elongation) rather than UTRs (affecting initiation), as the former allows fine-tuning of expression levels without blocking translation entirely.

An alternative explanation involves mRNA stability and localization. Coronavirus replication occurs in double-membrane vesicles derived from the endoplasmic reticulum, while translation of different structural proteins occurs at distinct cellular locations: Spike, Membrane, and Envelope proteins are translated on ER-associated ribosomes because of their transmembrane domains, whereas Nucleocapsid protein is translated on free cytosolic ribosomes (Sawicki et al., 2007; Snijder et al., 2003). G4 structures in S and N mRNA coding regions could serve as binding sites for RNA-binding proteins that facilitate mRNA localization to specific cytoplasmic compartments (ER membrane for S, cytosol for N), ensuring efficient virion assembly. The recent identification of G4-binding activity in SARS-CoV-2 Nsp3 (Lavigne et al., 2021)—a component of the replication-transcription complex—supports the hypothesis that viral proteins have evolved to recognize and interact with G4 structures within their own genome.

Host immune evasion may also contribute to this pattern. RNA structures including G4s can be recognized by pattern recognition receptors (PRRs) such as RIG-I and MDA5, triggering interferon responses (Métifiot et al., 2014). The localization of G4s specifically within protein-coding regions—rather than in highly conserved UTR regulatory elements—may represent an evolutionary compromise: maintaining G4-mediated regulatory functions while minimizing exposure of structured RNA in regions most critical for replication control. The complete absence of G4s in UTRs (0/31 genomes) supports strong selection against G4 formation in these functionally constrained regions.

Finally, evolutionary constraint provides additional support for functional G4 structures. Betacoronavirus conservation of G4 enrichment in S/N regions—maintained across lineages with divergence times spanning millions of years—indicates positive selection for functional G4 structures rather than neutral drift. This is further supported by the thermodynamic stability of S/N-localized G4s (57.9% of stable candidates) and the identification of a single highly conserved sequence (GGCTGGCAATGGCGG) present in 54.8% of genomes, exclusively in Nucleocapsid regions. Such deep conservation across diverse coronavirus lineages argues strongly for functional constraint maintaining these structures.

### 4.2 Host-virus interactions and G4 biology

The interplay between viral G4 structures and host cell machinery remains incompletely understood but offers additional mechanistic insights. Host cells express numerous G4-binding proteins, including helicases (DDX21, DHX36) and heterogeneous nuclear ribonucleoproteins (hnRNPs), which regulate cellular mRNA processing, localization, and translation (Varshney et al., 2020). Viral RNAs containing G4 structures could hijack these host factors to facilitate replication or translation, or conversely, viral proteins like Nsp3 could antagonize host G4-binding proteins to prevent antiviral responses.

The complete absence of G4s in coronavirus UTRs contrasts with their enrichment in S/N coding regions, suggesting that different selective pressures operate in different genomic contexts. UTRs contain highly conserved cis-acting elements essential for replication and translation (Sawicki et al., 2007); G4 formation in these regions could disrupt critical RNA-RNA or RNA-protein interactions, imposing strong negative selection. In contrast, coding regions allow greater structural flexibility, permitting G4 structures that do not disrupt reading frame or essential protein domains.

### 4.3 Therapeutic implications: from computational prediction to drug development

The potential therapeutic relevance of coronavirus G4 structures is supported by three convergent lines of evidence: (i) reported efficacy of G4-targeting small molecules against HIV-1 (Perrone et al., 2013a, 2013b) and influenza A (Majee et al., 2020; Wang et al., 2016), (ii) identification of G4-binding activity in SARS-CoV-2 Nsp3 (Lavigne et al., 2021) suggesting possible viral dependence on G4 recognition, and (iii) our computational identification of predicted thermodynamically stable, betacoronavirus-conserved G4 structures in functionally critical Spike and Nucleocapsid regions. These observations suggest the hypothesis that stabilizing G4 structures with small-molecule ligands may disrupt coronavirus replication, translation, or assembly, awaiting experimental confirmation.

Our computational analysis prioritizes the Nucleocapsid-localized sequence GGCTGGCAATGGCGG (predicted ΔG = -7.35 kcal/mol) as the primary candidate for experimental characterization and potential G4-ligand development. This sequence exhibits several computationally predicted properties favorable for therapeutic targeting. With respect to predicted thermodynamic favorability, ΔG = -7.35 kcal/mol suggests stable G4 formation under physiological conditions (37°C, 100 mM K${}^{+}$), with estimated melting temperature $T_{m}$ > 60°C, pending biophysical validation. Regarding betacoronavirus conservation, this sequence is present in 17 of 31 genomes (54.8%) spanning SARS-CoV-2 variants, SARS-CoV, MERS-CoV, and bat coronaviruses; this conservation across diverse betacoronavirus lineages is consistent with functional constraint and may indicate therapeutic potential across major pathogenic species. Concerning Nucleocapsid localization, the sequence is exclusively found in Nucleocapsid coding regions, targeting a protein essential for viral RNA packaging and assembly (V’kovski et al., 2021); if G4 formation is experimentally confirmed, disruption of Nucleocapsid function through G4 stabilization may inhibit virion production without requiring viral entry inhibition. Finally, druggability precedent exists because G4-stabilizing ligands (BRACO-19, PhenDC3, pyridostatin) show antiviral activity against HIV-1 and influenza with favorable therapeutic indices (Qin et al., 2022; Qin et al., 2023; Sun et al., 2025), providing chemical scaffolds for structure-activity relationship (SAR) studies optimizing selectivity for the target coronavirus G4 sequence.

The identified G4 candidates require experimental validation through staged approaches: first, biophysical confirmation of G4 formation using circular dichroism spectroscopy and thermal denaturation assays; second, ligand binding affinity assessment with established G4-stabilizing compounds (BRACO-19, PhenDC3, pyridostatin); and third, antiviral activity evaluation in cell-based viral replication assays to determine therapeutic index. Complete oligonucleotide synthesis specifications for the four prioritized candidates are provided in Supplementary Table S6A for immediate implementation by experimental researchers.

While the primary target sequence (GGCTGGCAATGGCGG) offers betacoronavirus-wide potential, three additional sequences show moderate conservation (3.2-9.7% prevalence) and could serve as secondary targets or for variant-specific applications. These include G4 motifs in Spike coding regions, which could offer distinct mechanisms of action (translation inhibition of the surface glycoprotein critical for viral entry) complementing Nucleocapsid-targeted ligands. A dual-target approach stabilizing G4s in both S and N coding regions could enhance therapeutic efficacy and reduce resistance emergence.

If confirmed experimentally, G4-targeted therapy may offer several advantages over conventional strategies. Target conservation represents a key advantage: G4 sequences show conservation across both coronavirus genera analyzed (Alphacoronavirus and Betacoronavirus), potentially reducing susceptibility to immune escape or rapid mutation compared to antibody-based therapies targeting variable epitopes. Furthermore, G4 conservation across betacoronavirus lineages may enable development of antivirals effective against SARS-CoV-2, SARS-CoV, MERS-CoV, and potentially novel emerging betacoronaviruses, contributing to preparedness for future outbreaks. G4 ligands may also complement existing antivirals (RNA polymerase inhibitors like remdesivir, protease inhibitors like nirmatrelvir) by targeting distinct mechanisms, potentially reducing effective doses and limiting resistance evolution. Finally, targeting structured RNA elements may constrain mutational escape, as maintaining G4 structure requires coordinated mutations across multiple guanine tracts, contrasting with protein-coding targets where single amino acid substitutions can confer resistance.

Should experimental validation confirm G4 formation and druggability of the prioritized candidates, medicinal chemistry optimization campaigns may be warranted to develop coronavirus-selective G4 ligands with favorable pharmacokinetic properties (oral bioavailability, tissue distribution, metabolic stability). Existing G4 ligands like quarfloxin (phase II clinical trials for cancer) provide precedent for safety and tolerability in clinical settings. The ongoing public health burden of COVID-19—combined with recurrent coronavirus emergence (SARS-CoV in 2003, MERS-CoV in 2012, SARS-CoV-2 in 2019)—highlights the value of exploring broad-spectrum coronavirus antivirals targeting conserved genomic features.

### 4.4 Methodological considerations

Our conservative consensus approach identified 137 G4 motifs across 31 coronavirus genomes (mean: 4.4 ± 1.5 per genome), fewer than the 512 potential candidates reported by Belmonte-Reche et al. (Belmonte-Reche et al., 2021) using relaxed detection criteria. This difference reflects deliberate methodological choices prioritizing specificity over sensitivity: (i) our consensus requirement (≥2 of 3 algorithms) reduces false positives compared to single-method detection, (ii) our G{2,3} pattern requires ≥2 contiguous guanines per tract versus relaxed criteria accepting single guanine runs, and (iii) our thresholds (G4Hunter ≥0.5, pqsfinder ≥20) emphasize canonical G4 structures over marginal candidates. While this conservative approach yields fewer total candidates, it ensures higher confidence for downstream rate ratio analysis and thermodynamic stability assessment, as marginal G4 predictions disproportionately contribute to zero-inflation and statistical instability. Our regional enrichment pattern (Spike: IRR=17.9; Nucleocapsid: IRR=15.2) aligns qualitatively with prior reports of S/N enrichment (Belmonte-Reche et al., 2021; Kusov et al., 2015; Lavigne et al., 2021), providing the first quantitative characterization using rigorous statistical methods. Importantly, our pipeline detected 100% (5/5) of experimentally validated SARS-CoV-2 G4 sequences from prior studies, including the RG-1 sequence confirmed by CD spectroscopy and in-cell NMR (Supplementary Table S8).

We initially attempted zero-inflated negative binomial (ZINB) regression on per-genome per-region counts (248 potential observations from 31 genomes × 8 regions, with 20 missing due to annotation gaps in some genomes, yielding 228 complete observations); however, with 85% zeros and small sample size (n=31), these models showed convergence failures and degenerate estimates (Supplementary Fig. S2). The extreme zero inflation provides limited information to distinguish structural zeros from sampling zeros, while n=31 is insufficient for stable estimation of ZINB’s multiple parameters per region (zero-inflation probability π, mean μ, and dispersion parameter α). We therefore adopted pooled Poisson rate ratios with region length as exposure as our primary analysis, yielding stable and interpretable incidence rate ratios (IRR). This approach aggregates G4 counts and base lengths across genomes by region before calculating rates, using ORF1ab as the reference region and computing ${\text{IRR}}_{r}=\left(c_{r}/e_{r}\right)/\left(c_{\text{ORF1ab}}/e_{\text{ORF1ab}}\right)$. The 95% confidence intervals were calculated using the score method as default, with Byar’s approximation and conditional exact methods reported in Supplementary Table S2 for sensitivity analysis.

For robustness verification, we also applied Poisson GLM with log(length) offset and robust standard errors, as well as quasi-Poisson models, obtaining consistent IRR estimates (Supplementary Table S2). Additionally, stratified analyses addressing pseudoreplication concerns (Supplementary Table S5) indicated that the paradoxical pattern is not an artifact of SARS-CoV-2 overrepresentation, with phylogenetically diverse subset analysis (n=11; Table S5) yielding comparable IRR estimates. The consistency across multiple estimation methods and sampling strategies strengthens confidence in the reported regional enrichment patterns.

Our phylogeny-aware analysis using generalized estimating equations (GEE) with genus-level clustering (Supplementary Table S7) provides partial accounting for phylogenetic structure. True generalized linear mixed models (GLMM) with random effects were attempted but showed instability given the small effective cluster sizes: our dataset comprises two genera (Alphacoronavirus n=2; Betacoronavirus n=29), with heavy overrepresentation of Betacoronavirus (primarily SARS-CoV-2 variants) creating imbalanced clusters unsuitable for stable random effects estimation. GEE with exchangeable correlation structure provides robust and consistent estimates under these conditions, with minimal within-genus correlation indicating limited phylogenetic clustering. The concordant IRRs across all strata (S and N both >10) and non-significant region × stratum interaction (p > 0.70) indicate that the paradoxical pattern of genome-wide depletion with marked regional retention does not depend on sampling composition. While the current sample size precludes full phylogenetic models, the consistency across independent species reinforces the biological validity of our findings.

### 4.5 Limitations and future directions

We acknowledge several limitations. G4 predictions are sequence-based and require experimental validation to confirm formation under physiological conditions, including effects of cellular RNA-binding proteins and translation complexes. Additionally, this analysis captures static G4 potential rather than dynamic formation during the viral lifecycle. Sample size considerations also warrant discussion: while n=31 enabled robust statistical testing through pooled Poisson methods and stratified analyses, larger datasets with balanced representation across coronavirus genera would strengthen phylogenetic inference. A methodological limitation concerns detection scope: although our consensus approach employs three complementary detection methods (regex, G4Hunter, pqsfinder), all three prioritize canonical G4 structures with standard loop lengths (1-7 nucleotides) and may underdetect non-canonical variants including bulge-containing G4s, long-loop G4s, and two-quartet structures that are increasingly recognized in viral RNA genomes (Bartas et al., 2019). This methodological choice, which prioritizes specificity over sensitivity, may result in false negatives for structurally atypical but biologically functional G4 motifs.

Future work should prioritize experimental validation of the identified betacoronavirus G4 candidates through biophysical characterization and antiviral efficacy testing, alongside mechanistic studies elucidating the functional roles of G4 structures in Spike and Nucleocapsid protein expression. Recent advances in G4-targeting compounds, including metallohelix-based ligands with enantioselective binding properties (Sun et al., 2025), and cross-virus applications demonstrating G4 targeting can enhance immune responses against diverse pathogens including monkeypox virus (Yang et al., 2025), provide promising directions for therapeutic development.

### 4.6 Code availability

Analysis code is provided as supplementary materials.

## 5 Conclusions

Through computational analysis of 31 coronavirus genomes using consensus G4 detection and robust statistical methods, we identified a betacoronavirus G4 distribution pattern characterized by moderate genome-wide depletion (mean FC=0.56) coupled with strong regional enrichment in Spike (IRR=17.9; 95% CI: 11.7-27.6) and Nucleocapsid (IRR=15.2; 95% CI: 8.7-26.6) proteins. This paradoxical pattern, validated through stratified sampling and multiple statistical methods (Supplementary Table S2), is consistent with opposing selection pressures: negative selection against G4-induced genomic instability in replication machinery regions, countered by functional selection for G4-mediated regulation in structural protein-coding regions. This pattern parallels observations in influenza A virus, suggesting convergent evolutionary constraints across taxonomically distant RNA viruses.

The consistent S/N protein enrichment across all coronavirus genera analyzed—combined with published evidence of G4 druggability in HIV-1, influenza, and SARS-CoV-2 (Bartas et al., 2019; Lavigne et al., 2021; Métifiot et al., 2014; Perrone et al., 2013; Perrone et al., 2013; Qin et al., 2022; Qin et al., 2023)—suggests these structurally conserved regions as potential candidates for broad-spectrum antiviral development, subject to experimental verification. Thermodynamic stability assessment identified 38 computationally predicted stable G4 candidates (ΔG < -5 kcal/mol), with 57.9% concentrated in S/N protein regions. Among these, the Nucleocapsid-localized sequence GGCTGGCAATGGCGG (predicted ΔG = -7.35 kcal/mol, 54.8% genome conservation) represents the primary candidate for experimental validation and potential G4-ligand development.

Our integrated computational framework combining sequence analysis, statistical modeling, and thermodynamic prediction provides a reproducible approach for generating hypothesis-driven therapeutic target candidates in emerging viral pathogens. The computational identification of conserved, predicted thermodynamically stable G4 structures in functionally critical coronavirus proteins suggests potential directions for structure-based antiviral strategies targeting genomic features rather than rapidly evolving epitopes. Should experimental validation confirm G4 formation and druggability, these findings may contribute to pandemic preparedness efforts and coronavirus therapeutic development.

## Declaration of generative AI and AI-assisted technologies in the writing process

The authors used Claude (Anthropic) for code development assistance and manuscript editing. The authors reviewed and edited all outputs and take full responsibility for the content of this publication.

## Data availability

All analysis code, genome sequences, and supplementary data are publicly available at Zenodo: https://doi.org/10.5281/zenodo.18295500. The repository includes Python scripts for G4 detection, statistical analysis, thermodynamic calculations, and all raw data required for complete reproduction of results.

## Declaration

All authors have read and agreed to the published version of the manuscript.

## CRediT authorship contribution statement

**Masato Tanigawa:** Writing – original draft, Software, Resources, Project administration, Methodology, Investigation, Formal analysis, Data curation, Conceptualization. **Takafumi Iwaki:** Writing – review & editing, Validation.

## Declaration of competing interest

The authors declare that they have no known competing financial interests or personal relationships that could have appeared to influence the work reported in this paper.

## Appendix Supplementary materials

Image, application 1

Image, application 2

Image, application 3

Image, application 4

Image, application 5

Image, application 6

Image, application 7

Image, application 8

Image, application 9

Image, application 10

Image, application 11

Image, application 12

Image, application 13

Image, application 14
