# Supplementary material for: Paradoxical G-quadruplex distribution in coronavirus genomes reveals functional constraints and antiviral therapeutic opportunities
Source: Virus Res. 2026 Jan 20;364:199692. doi: 10.1016/j.virusres.2026.199692 (PMC12860367; doi:10.1016/j.virusres.2026.199692)
Supplement: Supplementary file 1 [file mmc1.docx]

# Supplementary Figure Captions

## Figure S1. Forest Plot of Stratified IRR Analysis

**Forest plot showing consistent G4 enrichment across stratified analyses.** Incidence rate ratios (IRRs) with 95% confidence intervals are displayed for four analytical strata: (i) all genomes (n=31) using pooled Poisson rate ratios, (ii) one genome per species/lineage (n=11) to control for phylogenetic non-independence, (iii) SARS-CoV-2 variants only (n=20), and (iv) other coronaviruses only (n=11). Reference category is ORF1ab (IRR = 1.0, vertical dashed line). Both Spike (red diamonds) and Nucleocapsid (blue circles) proteins show consistent enrichment (IRR > 10) across all strata. The non-significant region × stratum interaction (Spike: p = 0.72; Nucleocapsid: p = 0.81) confirms that the paradoxical enrichment pattern is robust to sampling composition and not an artifact of SARS-CoV-2 overrepresentation.

## Figure S2. DHARMa-style Model Diagnostics for G4 Count Analysis

**Comprehensive diagnostic plots validating the aggregated Poisson modeling approach.** (A) Count distribution showing 82.7% zeros (282/341) in individual-level data (n=341 observations: 31 genomes × 11 regions). (B) Comparison of observed counts versus Poisson expectation, demonstrating severe zero-inflation. (C) Aggregated regional rates (G4 counts summed by region across all genomes), showing clear differentiation between regions. (D) Residual plot for individual-level Poisson model showing severe overdispersion. (E) Q-Q plot confirming non-normal residual distribution. (F) Aggregated model residuals for the saturated model (n=3 regions, df=0). (G) Zero-inflation test via simulation confirming significant excess zeros (p < 0.001). (H) Model comparison using Bayesian Information Criterion (BIC), with aggregated Poisson showing the best fit (lowest BIC = 89.2) while ZINB models failed to converge. (I) Summary statistics. Note: “Aggregated Poisson” refers to summing G4 counts and region lengths across genomes before calculating rate ratios, not meta-analytic pooling. Pearson dispersion statistics are not reported because the aggregated analysis uses a saturated model (3 regions with 3 parameters, yielding df=0), for which dispersion is mathematically undefined. Despite this limitation, aggregated rate ratios provide stable and interpretable estimates of regional G4 enrichment.
