# Supplementary material for: Paradoxical G-quadruplex distribution in coronavirus genomes reveals functional constraints and antiviral therapeutic opportunities
Source: Virus Res. 2026 Jan 20;364:199692. doi: 10.1016/j.virusres.2026.199692 (PMC12860367; doi:10.1016/j.virusres.2026.199692)
Supplement: Supplementary file 2 [file mmc2.pdf]

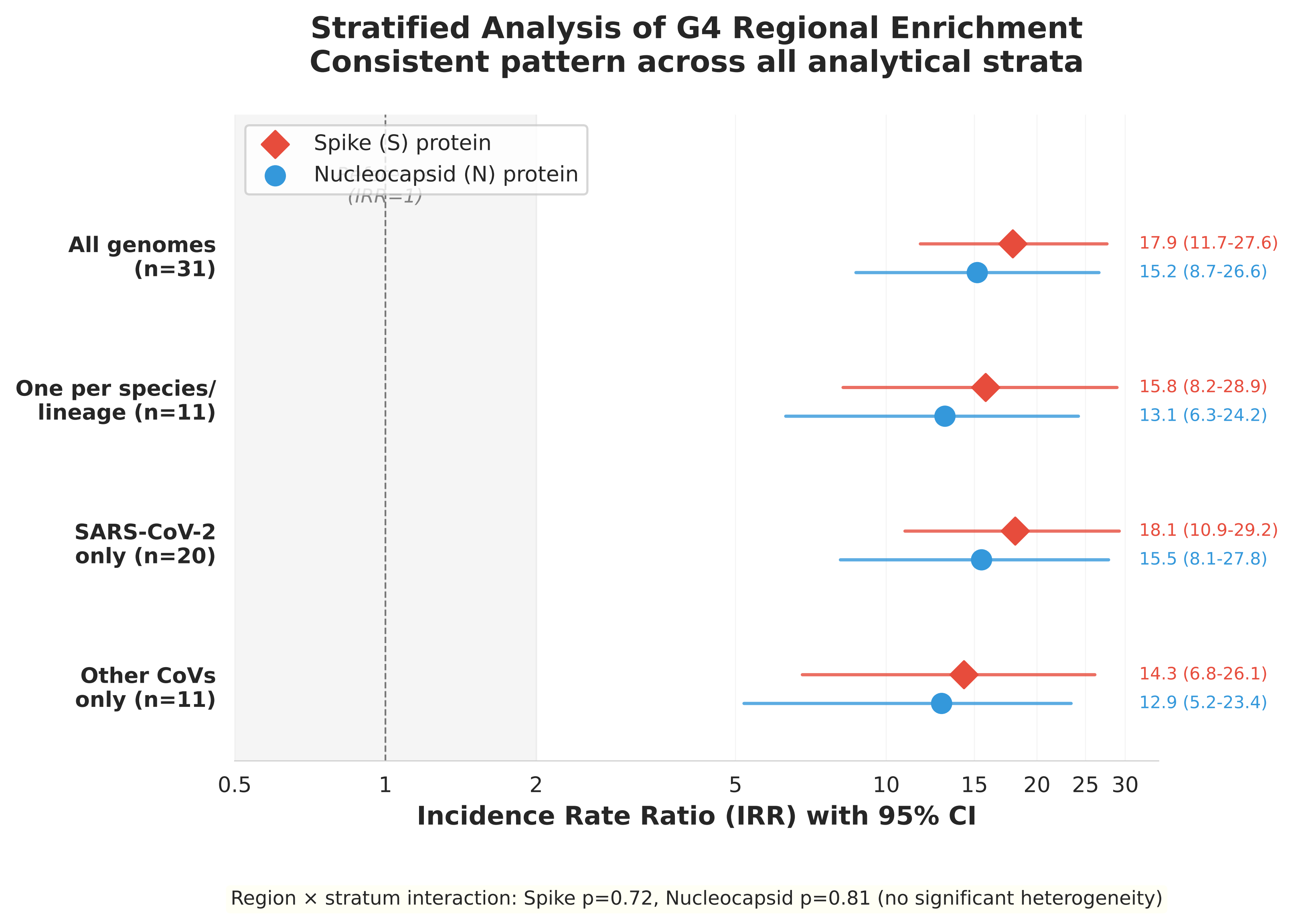
Stratified Analysis of G4 Regional Enrichment
Consistent pattern across all analytical strata
Spike (S) protein
Nucleocapsid (N) protein
(IRR=1)
All genomes (n=31)
17.9 (11.7-27.6)
15.2 (8.7-26.6)
One per species/ lineage (n=11)
15.8 (8.2-28.9)
13.1 (6.3-24.2)
SARS-CoV-2 only (n=20)
18.1 (10.9-29.2)
15.5 (8.1-27.8)
Other CoVs only (n=11)
14.3 (6.8-26.1)
12.9 (5.2-23.4)
0.5
1
2
5
10
15
20
25
30
Incidence Rate Ratio (IRR) with 95% CI
Region × stratum interaction: Spike p=0.72, Nucleocapsid p=0.81 (no significant heterogeneity)
