# Supplementary material for: Paradoxical G-quadruplex distribution in coronavirus genomes reveals functional constraints and antiviral therapeutic opportunities
Source: Virus Res. 2026 Jan 20;364:199692. doi: 10.1016/j.virusres.2026.199692 (PMC12860367; doi:10.1016/j.virusres.2026.199692)
Supplement: Supplementary file 5 [file mmc5.docx]

# Supplementary Table S3: Statistical Robustness of IRR Estimates

## Primary Analysis: Pooled Counts with Continuity Correction

| Region | Counts (c) | Exposure (e) | IRR (pooled) | 95% CI (Wald) | 95% CI (Score)† | 95% CI (Byar)§ | 95% CI (Exact)¶ |
| --- | --- | --- | --- | --- | --- | --- | --- |
| Spike | 87 | 118,482 | 17.9 | 11.7-27.6 | 11.7-27.6 | 11.5-27.2 | 11.6-28.1 |
| Nucleocapsid | 23 | 39,060 | 15.2 | 8.7-26.6 | 8.7-26.6 | 8.7-26.5 | 8.6-26.8 |
| ORF1ab (ref) | 27 | 659,990 | 1.0 | — | — | — | — |

Note: Counts are exact from raw data (no continuity correction needed for Wald and Score methods) †Score method (Wilson) confidence interval adapted for rate ratios [22] (using statsmodels implementation) §Byar’s approximation method for Poisson rate ratios (with +0.5 continuity correction) ¶Conditional exact confidence interval based on conditional hypergeometric distribution

## Confidence Interval Calculation Methods

### 1. Score Method (Wilson) [Primary]

The score method (Wilson confidence interval adapted for rate ratios) provides accurate coverage for Poisson rate ratios, even with small counts. This is the primary method used in the main manuscript [22].

### 2. Byar’s Approximation

Byar’s approximation (Breslow & Day, 1987) uses a log-transformation approach with continuity correction: - Add 0.5 to observed counts: c’_r = c_r + 0.5, c’_0 = c_0 + 0.5 - Variance of log(IRR): 1/c’_r + 1/c’_0 - 95% CI: exp(log(IRR’) ± 1.96√(1/c’_r + 1/c’_0))

The +0.5 continuity correction leads to slightly narrower confidence intervals compared to methods without correction (Spike: 11.5-27.2 vs 11.7-27.6).

### 3. Conditional Exact Method

Based on the conditional hypergeometric distribution given total counts (Lehmann, 1986). This method: - Makes no distributional assumptions beyond conditioning on total counts - Provides exact coverage probabilities - Results are nearly identical to score and Byar’s methods (11.6-28.1 vs 11.7-27.6 for Spike)

### 4. Wald Interval (for comparison)

Standard asymptotic method: IRR × exp{±1.96√(1/c_r + 1/c_0)} where c_r = region count, c_0 = reference count

## Consistency Across Statistical Methods

| Method | Spike IRR | 95% CI | Nucleocapsid IRR | 95% CI | Notes |
| --- | --- | --- | --- | --- | --- |
| **Pooled Poisson (Score)** | **17.9** | **11.7-27.6** | **15.2** | **8.7-26.6** | **Primary analysis** |
| Pooled Poisson (Score-log) | 17.9 | 12.1-29.7 | 15.2 | 8.3-26.9 | Log-scale score method |
| Poisson GLM (robust SE) | 17.8 | 11.5-27.4 | 15.1 | 8.6-26.5 | Sandwich estimator |
| Quasi-Poisson | 17.9 | 11.7-27.5 | 15.2 | 8.7-26.5 | Allows overdispersion |

## Additional Sensitivity Analyses

### Alternative Reference Regions

| Reference Region | Spike IRR | Nucleocapsid IRR | Notes |
| --- | --- | --- | --- |
| ORF1ab (primary) | 17.9 | 15.2 | Largest region, most stable |
| Genome-wide average | 15.3 | 13.0 | All regions pooled |
| Non-structural average | 16.5 | 14.0 | ORF1ab + ORF3a + accessory |

### Small Sample Adjustments

| Adjustment | Spike IRR | Nucleocapsid IRR | Notes |
| --- | --- | --- | --- |
| No adjustment | 17.9 | 15.2 | Raw counts (used in analysis) |
| Continuity correction (+0.5) | 17.5 | 15.0 | Alternative for sensitivity |
| Bayesian shrinkage | 17.2 | 14.8 | Empirical Bayes with weak prior |

## Model Comparison Statistics

| Model | AIC | BIC | Log-Likelihood | Convergence |
| --- | --- | --- | --- | --- |
| Pooled Poisson | 142.3 | 148.7 | -68.2 | Yes |
| Poisson GLM | 143.1 | 149.5 | -68.6 | Yes |
| Negative Binomial | 144.2 | 151.8 | -68.1 | Yes |
| ZINB (per-observation) | NaN | NaN | NaN | **No** |

## Interpretation

All robust statistical methods produce highly consistent IRR estimates: - **Spike protein**: IRR = 17.9 (95% CI range: 11.5-28.1 across all methods) - Wald method: 11.7-27.6 - Score method: 11.7-27.6 (identical to Wald for large samples) - Byar’s approximation: 11.5-27.2 (with +0.5 continuity correction) - Conditional exact: 11.6-28.1 (nearly identical to asymptotic methods) - **Nucleocapsid**: IRR = 15.2 (95% CI range: 8.6-26.8 across all methods) - Wald method: 8.7-26.6 - Score method: 8.7-26.6 (identical to Wald for large samples) - Byar’s approximation: 8.7-26.5 (with +0.5 continuity correction) - Conditional exact: 8.6-26.8 (nearly identical to asymptotic methods) - **ORF1ab**: IRR = 1.00 (reference region)

The remarkable consistency across four independent statistical methods (Wald, Score/Wilson, Byar’s, and conditional exact) strengthens confidence in the paradoxical pattern of regional enrichment despite genome-wide depletion. All confidence intervals overlap substantially, with maximum variation <10% (Spike: 11.5-28.1; Nucleocapsid: 8.6-26.8). This robustness is particularly important given the relatively modest sample sizes (23-87 G4 motifs per region), demonstrating that the findings are not sensitive to methodological assumptions about confidence interval calculation.

## Notes

1. **Data source**: 31 coronavirus genomes (20 SARS-CoV-2 + 11 other coronaviruses) from verified genome collections
2. **G4 detection**: Conservative pattern G{2,3}[ACTU]{1,15}G{2,3}[ACTU]{1,15}G{2,3}[ACTU]{1,15}G{2,3}
3. **ZINB failure**: Per-observation ZINB models failed to converge owing to sparse data
4. **Pooled approach**: Aggregating counts by region before analysis provides stable estimation
5. **Biological interpretation**: The 18-fold (Spike) and 15-fold (Nucleocapsid) enrichment in functional proteins remains biologically significant
