# Supplementary material for: Paradoxical G-quadruplex distribution in coronavirus genomes reveals functional constraints and antiviral therapeutic opportunities
Source: Virus Res. 2026 Jan 20;364:199692. doi: 10.1016/j.virusres.2026.199692 (PMC12860367; doi:10.1016/j.virusres.2026.199692)
Supplement: Supplementary file 8 [file mmc8.docx]

# Supplementary Table S5: Stratified Analyses Addressing Pseudoreplication (CORRECTED)

## Label Correction Notice

**Labeling error corrected**: MN975262.1 was incorrectly labeled as “Bat coronavirus RmYN02” in figures and tables of the original submission. Upon NCBI verification, this sequence is SARS-CoV-2 isolate 2019-nCoV_HKU-SZ-005b_2020 (Hong Kong early isolate).

**Important**: This was a **labeling error only**. Sample counts were always correct in the underlying data: - SARS-CoV-2 variants: n=20 - Other coronaviruses: n=11 - Total: n=31

**Note**: Bat coronavirus RmYN02 (EPI_ISL_412977) is available only on GISAID, not NCBI, and was never included in this NCBI-based dataset.

## Analysis Strategy

To address potential pseudoreplication from the overrepresentation of SARS-CoV-2 variants (20/31 genomes), we performed stratified analyses with consistent methodology. All analyses used pooled Poisson rate ratios with score-based confidence intervals.

## Table S5: Incidence Rate Ratios by Analysis Stratum

| Analysis Stratum | n | Spike (S) IRR (95% CI) | Nucleocapsid (N) IRR (95% CI) | Justification |
| --- | --- | --- | --- | --- |
| **All genomes** | 31 | 17.9 (11.7-27.6) | 15.2 (8.7-26.6) | Primary analysis, complete dataset |
| **Phylogenetically diverse subset**¹ | 11 | 15.8 (8.2-28.9)² | 13.1 (6.3-24.2)² | Maximizes phylogenetic diversity, minimizes pseudoreplication |
| **SARS-CoV-2 variants only** | 20 | 18.1 (10.9-29.2)² | 15.5 (8.1-27.8)² | Within-species consistency |
| **Other coronaviruses only** | 11 | 14.3 (6.8-26.1)² | 12.9 (5.2-23.4)² | Cross-species validation |

**Reference category**: ORF1ab (IRR = 1.0) **Method**: Pooled Poisson rate ratios, IRR = (c_r/e_r)/(c_0/e_0) where c = counts, e = exposure (region length) **95% CI**: Score method (Wilson score interval for rate ratios)

## Notes

¹ **Phylogenetically diverse subset selection**: To address pseudoreplication from multiple closely related SARS-CoV-2 variants, we selected 11 genomes maximizing phylogenetic diversity within our dataset: - **SARS-CoV-2**: NC_045512.2 (Wuhan-Hu-1 reference genome) - **Human coronaviruses** (6 lineages): SARS-CoV-1 (AY291451.1), MERS-CoV (JX869059.2), HCoV-229E (AF304460.1), HCoV-NL63 (AY567487.2), HCoV-OC43 (AY585228.1), HCoV-HKU1 (AY597011.2) - **Bat sarbecoviruses** (2 isolates): RaTG13 (MN996532.2, Yunnan 2013), BANAL-52 (MZ937000.1, Laos 2020) - **Pangolin sarbecoviruses** (2 isolates): GX-P4L (MT040333.1, Guangdong), GX-P2V (MT072864.1, Guangxi)

**Taxonomic note**: Under current ICTV classification, SARS-CoV-2, RaTG13, BANAL-52, and pangolin coronaviruses are all classified within the same species (*Severe acute respiratory syndrome-related coronavirus*, subgenus *Sarbecovirus*). Our selection criteria prioritized **statistical independence** (different host species, geographic origins, and phylogenetic positions) rather than strict taxonomic species boundaries, which is appropriate for addressing pseudoreplication in comparative genomic analyses.

² Values estimated from subset analyses. Exact values may vary slightly depending on genome availability in the dataset.

³ **Sample size clarification**: The four strata are: (i) All genomes (n=31) = 20 SARS-CoV-2 + 11 other coronaviruses; (ii) Phylogenetically diverse subset (n=11) = 11 genomes representing major coronavirus lineages (see note ¹); (iii) SARS-CoV-2 only (n=20); (iv) Other coronaviruses only (n=11).

## Statistical Robustness

### Model Diagnostics

- **Convergence**: All models converged successfully
- **Zero inflation**: UTRs excluded owing to absence of G4s (0/31 genomes; 95% CI: 0-9.7% by rule of three)
- **Dispersion**: Note: Pearson dispersion statistics are not applicable for the pooled analysis because it uses a saturated model (3 regions with 3 parameters, yielding df=0)
- **Model selection**: BIC_NB = 142.3 < BIC_Poisson = 148.7 < BIC_ZIP = 154.2 (ZINB failed to converge)
- **Goodness of fit**: R²(marginal) = 0.42 (based on fixed effects)

### Sensitivity Analyses

- **Alternative CI methods**: Wald method yielded similar results (Spike: 11.5-27.4; Nucleocapsid: 8.6-26.5)
- **Genus-level clustering**: GEE with exchangeable correlation structure confirmed findings (Supplementary Table S7). True GLMMs with genus random effects were attempted but showed instability owing to small cluster sizes (n=2 genera, highly imbalanced). GEE with exchangeable working correlation provides robust estimates under these conditions.

## Interaction Testing

To formally test whether IRRs differ across strata, we fitted a combined model:

count ~ region * stratum + offset(log(length))

**Results**: - Region × stratum interaction for Spike: χ² = 1.32, p = 0.72 - Region × stratum interaction for Nucleocapsid: χ² = 0.94, p = 0.81

No significant differences in IRRs across strata (both p > 0.70), confirming that the paradoxical pattern is consistent regardless of sampling strategy.

## Interpretation

1. **Consistency across strata**: All analyses show S and N enrichment (IRR > 10) relative to ORF1ab
2. **Robustness to pseudoreplication**: Phylogenetically diverse subset analysis (n=11) confirms the pattern with statistical independence
3. **Generalizability**: Pattern holds within SARS-CoV-2 variants and across coronavirus genera
4. **Statistical validity**: Results are not artifacts of SARS-CoV-2 oversampling

## Conclusion

The stratified analyses demonstrate that the paradoxical G4 distribution pattern—genome-wide depletion coupled with regional enrichment in S and N proteins—is a robust biological phenomenon rather than a statistical artifact of sampling bias.

## Summary of Label Correction

**Nature of error**: MN975262.1 was incorrectly labeled as “Bat coronavirus RmYN02” in figure and table legends. This was a labeling error only; sample counts were always correct (20 SARS-CoV-2 + 11 other = 31 total).

| Item | Correction |
| --- | --- |
| MN975262.1 label | “Bat coronavirus RmYN02” → “SARS-CoV-2 HKU-SZ-005b_2020” |
| Bat coronavirus list | Removed RmYN02 reference (GISAID-only, never in dataset) |

**Sample counts**: Unchanged (always 20 SARS-CoV-2 + 11 other coronaviruses = 31 total) **IRR values**: Unchanged (Spike: 17.9, Nucleocapsid: 15.2)

*Corrected for VIRUS-D-25-00454 revision* *Date: 2026-01-04*
