# Supplementary material for: Paradoxical G-quadruplex distribution in coronavirus genomes reveals functional constraints and antiviral therapeutic opportunities
Source: Virus Res. 2026 Jan 20;364:199692. doi: 10.1016/j.virusres.2026.199692 (PMC12860367; doi:10.1016/j.virusres.2026.199692)
Supplement: Supplementary file 9 [file mmc9.docx]

# Table S6A: S/N Region G4 Candidate Set for Experimental Validation

**Supplementary Table S6A. Experimentally validated G4 candidate set from Spike/Nucleocapsid protein regions.**

This table presents the 4 unique G4 sequences found within the 22 thermodynamically stable instances (ΔG < -5 kcal/mol) located in functionally critical Spike and Nucleocapsid protein regions. Sequences are prioritized based on conservation rates across 31 coronavirus genomes, with Priority 1 candidates (≥50% conservation) recommended for Phase 1 experimental validation targeting pan-coronavirus therapeutic development, and variant-specific candidates (<10% conservation) suitable for Phase 2 specialized applications.

**Column Descriptions:** - **Unique_Sequence**: G4-forming DNA sequence (5’ to 3’) - **Protein_Region**: SARS-CoV-2 protein domain (Spike: 21563-25384, Nucleocapsid: 28274-29533) - **Instance_Count**: Number of instances across all stable S/N region G4s (total = 22) - **Conservation_Rate_Percent**: Percentage of genomes containing this sequence (n=31 genomes) - **Mean_dG_kcal_mol**: Thermodynamic stability at 37°C (more negative = more stable) - **GC_Content_Percent**: G+C nucleotide content - **Therapeutic_Priority**: Pan-coronavirus target classification based on conservation - **Experimental_Phase**: Recommended validation phase (Phase 1: immediate priority) - **Representative_Coordinates**: SARS-CoV-2 reference genome coordinates (NC_045512.2) - **Oligonucleotide_5to3**: RNA oligonucleotide for synthesis (DNA→RNA: T→U) - **Expected_Tm_C**: Predicted melting temperature in KCl buffer

**Experimental Recommendations:** - **Primary Target (Priority 1)**: GGCTGGCAATGGCGG - Phase 1 validation using CD spectroscopy, thermal denaturation, and ligand binding assays (pyridostatin, BRACO-19, PhenDC3) - **Secondary Targets (Variant-specific)**: Phase 2 validation for specialized applications or backup therapeutic development - **Buffer Conditions**: 10 mM Tris-HCl (pH 7.4), 100-150 mM KCl, 1 mM MgCl₂, 37°C - **Success Criteria**: G4 formation confirmation (CD signature), Tm >55°C, ligand binding (IC₅₀ <50 μM)

This candidate set provides immediately actionable data for G4-targeting antiviral drug discovery, with complete experimental protocols detailed in the main manuscript Methods section 2.4.3.
