# Supplementary material for: Paradoxical G-quadruplex distribution in coronavirus genomes reveals functional constraints and antiviral therapeutic opportunities
Source: Virus Res. 2026 Jan 20;364:199692. doi: 10.1016/j.virusres.2026.199692 (PMC12860367; doi:10.1016/j.virusres.2026.199692)
Supplement: Supplementary file 12 [file mmc12.docx]

# Supplementary Table S7: Complete GEE Model Results

## Model Specification

**Formula**: log(E[count]) = β₀ + β_S·I(S) + β_N·I(N) + β_GC·GC + β_size·Size + log(length)

Where: - I(S), I(N): Indicator variables for Spike and Nucleocapsid regions (reference: ORF1ab) - GC: GC content (%) - Size: Genome size (nucleotides) - log(length): Offset for region length

**Method**: Generalized Estimating Equations (GEE) with exchangeable correlation structure and genus-level clustering. GEE was used as an approximation to GLMM because true mixed models were unstable owing to small cluster sizes (n=2 genera, highly imbalanced).

## Fixed Effects Estimates

| Parameter | Coefficient | SE | z-value | p-value | 95% CI | IRR | IRR 95% CI |
| --- | --- | --- | --- | --- | --- | --- | --- |
| Spike (β_S) | 2.936 | 0.281 | 10.45 | <0.001 | (2.385, 3.486) | 18.84 | (10.86, 32.66) |
| Nucleocapsid (β_N) | 2.763 | 0.021 | 133.99 | <0.001 | (2.723, 2.803) | 15.85 | (15.22, 16.50) |
| GC Content (β_GC) | 0.009 | 0.021 | 0.45 | 0.65 | (-0.031, 0.050) | — | — |
| Genome Size (β_size) | -0.0002 | 0.00002 | -11.16 | <0.001 | (-0.0003, -0.0001) | — | — |

## Interpretation

### Regional Effects (Primary Findings)

- **Spike protein (β_S)**: Highly significant enrichment relative to ORF1ab (p < 0.001)
- **Nucleocapsid (β_N)**: Highly significant enrichment relative to ORF1ab (p < 0.001)
- **IRR interpretation**: 17-18× higher G4 density in Spike, 15-16× higher in Nucleocapsid vs ORF1ab

### Compositional Covariates (Biologically Non-Significant)

- **GC content (β_GC)**: Not statistically associated with G4 density (β = 0.009, p = 0.65). The effect size is negligible and non-significant.
- **Genome size (β_size)**: Statistically significant (p < 0.001) but biologically negligible (β = -0.0002, effect size < 0.01%). The coefficient is three orders of magnitude smaller than regional effects and represents no meaningful biological relationship.
- **Biological interpretation**: The paradoxical G4 distribution pattern (regional enrichment despite genome-wide depletion) is not explained by compositional biases. The 19-fold enrichment in Spike and 16-fold enrichment in Nucleocapsid (compared to ORF1ab) far exceed any compositional effects (GC or genome size), confirming that regional enrichment represents functional selection rather than compositional artifact.

### Genus Clustering

- **Correlation structure**: Exchangeable working correlation for genus-level clustering
- **Within-genus correlation**: Minimal correlation indicates limited phylogenetic clustering
- **Interpretation**: G4 enrichment pattern is consistent across Alphacoronavirus and Betacoronavirus genera

## Model Diagnostics

- **Convergence**: Model converged successfully
- **Sample size**: n=93 region observations from 31 genomes (20 SARS-CoV-2, 11 other coronaviruses)
- **Genera**: Betacoronavirus (n=29), Alphacoronavirus (n=2)
- **Correlation structure**: Exchangeable working correlation for genus clustering
- **Overdispersion**: Negative binomial family accounts for count overdispersion

## Notes

1. **GEE vs GLMM**: We used GEE as a robust approximation to GLMM. True GLMMs with genus random effects were attempted but unstable owing to small cluster sizes and severe imbalance (29 vs 2 genomes).
2. **Score-based inference**: Primary IRR estimates in main text use pooled Poisson with score-based confidence intervals for maximum accuracy with small counts (Spike: 17.9, 95% CI 11.7-27.6; Nucleocapsid: 15.2, 95% CI 8.7-26.6). GEE results provided here for completeness and sensitivity analysis.
3. **Nucleocapsid confidence interval**: The narrow GEE confidence interval for Nucleocapsid (15.22-16.50) compared to the primary analysis (8.7-26.6) reflects GEE’s use of the robust sandwich estimator and genome size covariate adjustment. The primary pooled Poisson confidence intervals (score method) are more conservative and recommended for count data with high zero-inflation. Both methods confirm strong Nucleocapsid enrichment (IRR >10).
4. **Compositional covariates**: GC content and genome size were included to rule out compositional explanations for the paradoxical pattern. Their non-significance strengthens the interpretation that regional enrichment represents functional selection rather than compositional artifact.
5. **Publication bias consideration**: We report all model coefficients (including non-significant covariates) for transparency and to avoid selective reporting bias.
