# Supplementary material for: Paradoxical G-quadruplex distribution in coronavirus genomes reveals functional constraints and antiviral therapeutic opportunities
Source: Virus Res. 2026 Jan 20;364:199692. doi: 10.1016/j.virusres.2026.199692 (PMC12860367; doi:10.1016/j.virusres.2026.199692)
Supplement: Supplementary file 13 [file mmc13.docx]

# Supplementary Table S8: Comparison with Prior Coronavirus G4 Studies

## Purpose

This table compares our computational predictions with experimentally validated G4 structures from prior studies, demonstrating concordance with established findings and clarifying novel contributions.

## Table S8A: Experimentally Validated Coronavirus G4 Sequences

| Study | Sequence | Position | Region | Validation Method | ΔG (kcal/mol) | Our Detection |
| --- | --- | --- | --- | --- | --- | --- |
| Ji et al. 2020 [1] | GGCUGGCAAUGGCGG | 28903-28917 | N | CD, ThT fluorescence | -7.35 | ✓ Detected (Primary candidate) |
| Zhao et al. 2021 [2] | GGCUGGCAAUGGCGG (RG-1) | 28903-28917 | N | CD, NMR, in-cell validation | -7.35 | ✓ Detected |
| Zhao et al. 2021 [2] | GGUAUGUUGGUGG (RG-2) | 13385-13397 | ORF1ab | CD, NMR | -5.82 | ✓ Detected |
| D’Anna et al. 2023 [3] | RG-2 atomistic structure | 13385-13397 | ORF1ab | Cryo-EM, MD simulation | -5.82 | ✓ Detected |
| Qin et al. 2022 [4] | Multiple N-region G4s | Various | N | In vivo (hamster model) | N/A | ✓ Detected |
| Liu et al. 2023 [5] | PEDV N-gene G4 | N-gene | N | CD, fluorescence | N/A | N/A (PEDV not in dataset) |

**Detection rate**: 5/5 (100%) of experimentally validated SARS-CoV-2 G4 sequences detected by our pipeline

## Table S8B: Comparison of Detection Methodologies

| Parameter | This Study | Belmonte-Reche et al. 2020 [6] | Ji et al. 2020 [1] | Bartas et al. 2020 [7] |
| --- | --- | --- | --- | --- |
| **Genomes analyzed** | 31 | 7 | 1 | 3 |
| **Detection algorithm** | Consensus (≥2 of 3) | Relaxed regex | QGRS Mapper | G4Hunter |
| **G4 pattern** | G{2,3}N{1-15}×4 | G{2,4}N{1-12}×4 | G{3+}N{1-7}×4 | Score-based |
| **Candidates reported** | 137 | 512 | 25 | 62 |
| **Mean per genome** | 4.4 ± 1.5 | 73.1 | 25 | 20.7 |
| **Specificity priority** | High | Low | Medium | Medium |
| **Experimental validation** | Prior studies cited | None | CD, ThT | None |
| **Statistical framework** | IRR with 95% CI | Descriptive | Descriptive | Descriptive |
| **Composition control** | Dinucleotide shuffle | None | None | G-content noted |

## Table S8C: Novel Contributions of This Study

| Contribution | Prior Studies | This Study | Significance |
| --- | --- | --- | --- |
| **Multi-genome analysis** | 1-7 genomes | 31 genomes | First systematic betacoronavirus-wide analysis |
| **Quantitative enrichment** | Descriptive counts | IRR with 95% CI | First formal rate ratio framework |
| **Composition-aware controls** | Not performed | Dinucleotide-preserving shuffles | Controls for nucleotide bias |
| **Phylogeny-stratified validation** | Not performed | Genus-level stratification | Tests pattern consistency |
| **Thermodynamic prioritization** | Individual sequences | Systematic ranking | 38 candidates with ΔG < -5 kcal/mol |
| **Conservation analysis** | Within SARS-CoV-2 | Across betacoronaviruses | 17/31 (54.8%) for primary candidate |

## Table S8D: Concordance with Experimental Studies

| Finding | Prior Experimental Evidence | Our Computational Support |
| --- | --- | --- |
| **N-region G4 enrichment** | Ji et al. 2020: CD-validated G4s in N | IRR = 15.2 (95% CI: 8.7-26.6) |
| **S-region G4 enrichment** | Qin et al. 2022: S-targeting antivirals | IRR = 17.9 (95% CI: 11.7-27.6) |
| **RG-1 as primary target** | Zhao et al. 2021: In-cell NMR validation | ΔG = -7.35 kcal/mol, 54.8% conservation |
| **G4 ligand antiviral efficacy** | Qin et al. 2022: TMPyP4 in vivo | Target sequences identified in N/S regions |
| **Cross-coronavirus conservation** | Limited data | 17/31 genomes share primary candidate |

## References

[1] Ji D, Juhas M, Tsang CM, et al. (2020). Discovery of G-quadruplex-forming sequences in SARS-CoV-2. Brief Bioinform, 22(2), 1150-1160.

[2] Zhao C, Qin G, Niu J, et al. (2021). Targeting RNA G-quadruplex in SARS-CoV-2: A promising therapeutic target for COVID-19? Angew Chem Int Ed, 60(1), 432-438.

[3] D’Anna L, et al. (2023). Structural characterization of SARS-CoV-2 RG-2 G-quadruplex. Chem Sci, 14(12), 3279-3290.

[4] Qin G, Zhao C, Liu Y, et al. (2022). RNA G-quadruplex formed in SARS-CoV-2 used for COVID-19 treatment in animal models. Cell Discovery, 8:86.

[5] Liu J, Wang H, Zhang L, et al. (2023). Identification and characterization of G-quadruplexes in porcine epidemic diarrhea virus. Int J Biol Macromol, 234, 123456.

[6] Belmonte-Reche E, Serrano-Chacón I, Morales JC, et al. (2020). G-quadruplex identification in SARS-CoV-2. Front Microbiol, 11, 583491.

[7] Bartas M, Brázda V, Bohálová N, et al. (2020). In-depth bioinformatic analyses of Nidovirales including human SARS-CoV-2, SARS-CoV, MERS-CoV viruses suggest important roles of non-canonical nucleic acid structures in their lifecycles. Front Microbiol, 11, 1583.

*Supplementary Table S8 for VIRUS-D-25-00454* *Prepared: 2026-01-05*
