# Supplementary material for: Paradoxical G-quadruplex distribution in coronavirus genomes reveals functional constraints and antiviral therapeutic opportunities
Source: Virus Res. 2026 Jan 20;364:199692. doi: 10.1016/j.virusres.2026.199692 (PMC12860367; doi:10.1016/j.virusres.2026.199692)
Supplement: Supplementary file 14 [file mmc14.docx]

# Supplementary Table S9: Composition-Aware G4 Enrichment Analysis

## Purpose

This analysis addresses Reviewer #2’s critical methodological concern: “Enrichment could be driven by G-content rather than selection for G4 formation. Please add region-wise expected counts using dinucleotide-preserving shuffles.”

## Table S9A: Regional Nucleotide Composition (Reference: NC_045512.2)

| Region | Length (bp) | G-content (%) | A (%) | T (%) | C (%) |
| --- | --- | --- | --- | --- | --- |
| ORF1ab | 21,289 | 19.9 | 29.8 | 32.1 | 18.2 |
| Spike | 3,821 | 18.4 | 30.2 | 33.5 | 17.9 |
| Nucleocapsid | 1,259 | 22.2 | 28.1 | 31.4 | 18.3 |

**Note**: G-content is similar across regions (18.4-22.2%), with Spike having the *lowest* G-content.

## Table S9B: Observed vs Expected G4 Counts (Composition-Matched Simulation)

| Region | Observed G4/genome | Expected G4¹ | Fold Change | P-value² | Interpretation |
| --- | --- | --- | --- | --- | --- |
| ORF1ab | 0.87 | 5.95 | **0.15** | 0.034* | Significant depletion |
| Spike | 2.81 | 0.60 | **4.66** | 0.004** | Significant enrichment |
| Nucleocapsid | 0.74 | 0.67 | 1.10 | 0.93 | No significant difference |

¹ Expected G4 counts calculated from 1,000 random sequences per region with matched G-content (mononucleotide-preserving shuffle) ² Two-tailed z-test; * p < 0.05, ** p < 0.01

## Table S9C: G-Content Adjusted Incidence Rate Ratios

| Region | Raw IRR (vs ORF1ab) | G-content Adjustment Factor¹ | Adjusted IRR |
| --- | --- | --- | --- |
| ORF1ab | 1.0 (reference) | 1.0 | 1.0 |
| Spike | 17.9 | 1.08 | **19.4** |
| Nucleocapsid | 15.2 | 0.90 | **13.7** |

¹ Adjustment factor = G-content(ORF1ab) / G-content(region)

**Key finding**: After G-content adjustment, Spike and Nucleocapsid still show >10-fold enrichment, confirming that the regional enrichment pattern is NOT an artifact of nucleotide composition.

## Methodology

### Composition-Matched Null Model

For each region, we generated 1,000 random sequences with identical: - Sequence length - Mononucleotide composition (G, A, T, C percentages)

G4 motifs were detected using the pattern: G{2,4}[ATGC]{1,7}G{2,4}[ATGC]{1,7}G{2,4}[ATGC]{1,7}G{2,4}

### Statistical Testing

- **Fold change** = Observed / Expected
- **P-value** calculated using z-test: z = (Observed - Expected) / SD(Expected)
- **IRR adjustment** normalizes for regional G-content differences

## Key Conclusions

1. **Spike enrichment cannot be explained by G-content**: Despite having the *lowest* G-content (18.4%), Spike shows the *highest* G4 enrichment (fold-change = 4.66, p = 0.004).
2. **ORF1ab depletion is genuine**: ORF1ab contains significantly fewer G4s than expected by chance (fold-change = 0.15, p = 0.034), suggesting active selection against G4 formation in the replication machinery.
3. **Nucleocapsid enrichment reflects rate ratio, not composition**: While composition-matched simulation shows no significant enrichment for Nucleocapsid (fold-change = 1.10), the high IRR (15.2) reflects enrichment *relative to ORF1ab*, which is itself depleted.
4. **Regional enrichment reflects functional constraints**: The composition-aware analysis confirms that the paradoxical G4 distribution pattern (genome-wide depletion with regional enrichment in S and N) represents genuine biological selection, not nucleotide composition artifacts.

## Data Availability

Analysis script: composition_aware_analysis.py Raw output: composition_aware_enrichment.csv

*Supplementary Table S9 for VIRUS-D-25-00454* *Analysis method: Mononucleotide-preserving shuffle simulation (n=1000 per region)* *Prepared: 2026-01-05*
